# Achieving Achromatic and Misalignment-Tolerant Fiber Coupling via Meta-Lens with Structural Interleaving

**DOI:** 10.3390/nano16090557

**Published:** 2026-05-01

**Authors:** Xinlie Yuan, Zhenhuan Tian, Ben Jia, Yong Zhang, Yong Zhou, Changfei Hu, Qijian Xu, Feng Yun

**Affiliations:** 1 Shaanxi Provincial Key Laboratory of Photonics & Information Technology, Xi’an Jiaotong University, Xi’an 710049, China; yuanxinlie@stu.xjtu.edu.cn (X.Y.); jiabeng@stu.xjtu.edu.cn (B.J.); 2 Wuhan Huagong Genuine Optics Technology Co., Ltd., Wuhan 430223, China; zhangyong@genuine-opto.com (Y.Z.); zhouyong@genuine-opto.com (Y.Z.); roberthu@genuine-opto.com (C.H.); xuqijian@genuine-opto.com (Q.X.)

**Keywords:** meta-lens, achromatic design, particle swarm optimization, structural interleaving, anti-displacement performance, phase matching

## Abstract

This paper addresses the chromatic aberration and off-axis collimation issues in the laser–lens–fiber coupling system by proposing a chromatic aberration-corrected Meta-lens design based on a particle swarm optimization algorithm and structural interleaving method. By establishing an optimization model that includes wavelength-dependent phase factors, achromatic performance with a focal length standard deviation of less than 0.4 μm is achieved in the 1260–1360 nm band. Innovatively, the structural interleaving technique is adopted to integrate multiple different phase distributions into a single meta-surface, keeping the coupling efficiency fluctuation within 8% over a ±1 μm off-axis displacement range. The research results demonstrate that this method effectively solves the phase quantization and dispersion matching challenges of large-scale meta-lens, achieving a phase matching efficiency of 95.2%, providing a feasible path for the engineering application of highly robust meta-lens in high-precision optical systems.

## 1. Introduction

The laser–lens–fiber coupling system is a critical assembly whose optical lens position stability directly determines system efficiency and reliability, especially in high-speed optical communications, LiDAR, silicon photonics integration, and the biomedical imaging domain [1,2,3,4]. The mode field diameter of the single-mode fiber is usually ~10 μm in a typical laser–lens–single-mode fiber coupling optical system, which making the system highly sensitive to beam profile, focal length accuracy, and spatial alignment [5,6]. Even minute lens misalignments—caused by manufacturing tolerances, assembly errors, system vibrations, or thermal expansion—can significantly degrade coupling efficiency and quality [7,8]. Moreover, lens chromatic aberration causes wavelength-dependent focal shifts, resulting in inconsistent coupling efficiencies across multiple channels and thereby compromising system uniformity [9,10]. Therefore, achieving stable optical power delivery and high-reliability tolerance under lens micro-displacement disturbances has become an urgent key challenge [11].

To improve coupling tolerance, researchers have conducted extensive explorations from multiple directions, such as the fiber end, optical path structure, and coupling methods. For instance, by using beam-expanding fibers, multi-core fibers, or few-mode fibers, the effective receiving aperture is increased, thereby reducing sensitivity to the position and angle of the incident beam [12,13]. Some schemes introduce collimators, micro-lens arrays, or self-focusing elements into the optical path to improve beam divergence and enhance tolerance to displacement perturbations [14]. Additionally, studies have attempted to compensate for device drift through mechanical self-alignment structures or active feedback locking methods [15]. These methods have improved coupling system stability to some extent, but they often suffer from issues such as complex structures, increased device volume, high manufacturing costs, or stringent requirements for system compatibility. Meanwhile, many schemes primarily focus on improving the fiber end mode distribution or system-level optical paths [16], without addressing the fundamental contradiction between chromatic aberration and displacement sensitivity through the optical modulation capabilities of the lens itself. There is a lack of an effective design approach that can simultaneously achieve broadband achromatic performance and high displacement tolerance within a single lens.

A meta-lens consists of nanoscale structures at subwavelength dimensions. Each structural unit can independently modulate the phase, amplitude, and polarization of transmitted light through precise geometric design, enabling pixel-level customization of the wavefront [17,18,19,20]. By assigning customized phase distributions to different incident positions, a meta-lens can integrate multiple optical functions into a single physical device, allowing the lens to automatically activate corresponding local modulation regions upon micro-displacement, thereby compensating for wavefront perturbations [21]. This inherent property makes meta-lenses naturally suitable for anti-lens-displacement perturbation design, significantly reducing the impact of lens position variation on far-field collimation effects and coupling efficiency, while providing structural-level assurance for stable output in optical systems [22]. Furthermore, by combining optimization algorithms with phase library expansion strategies, not only can the phase discontinuity issues commonly encountered when scaling the angular spectrum method to large-size meta-lenses be resolved, but chromatic aberration compensation and displacement robustness can also be simultaneously achieved on a single device [23,24,25]. This offers a novel, high-stability solution for laser–lens–fiber coupling systems. Based on this concept, this paper proposes a meta-lens design method that simultaneously achieves broadband achromatic performance and anti-lens-displacement perturbation characteristics, providing a new technical pathway to enhance the tolerance of coupling links.

Therefore, this study presents an engineering-feasible meta-lens design that simultaneously achieves broadband achromaticity, displacement robustness, and large-scale phase matching. Broadband performance is achieved by employing a wavelength-dependent compensation phase model combined with particle swarm optimization, which facilitates the collaborative multi-wavelength design over the 1260–1360 nm band while minimizing focal drift [26,27,28,29,30]. Displacement tolerance is enhanced via structural interleaving, which regionally integrates phase profiles for 0 and ±1 μm off-axis shifts into a single meta-surface, maintaining stable focusing within ±1 μm displacement [31]. Large-aperture applicability is addressed through mixed parametric scanning of multiple nanostructures, expanding phase coverage and achieving >95% phase-matching efficiency [31,32]. Validated by multi-scale FDTD and angular spectrum simulations, this framework offers a manufacturable path toward meta-lenses with superior broadband consistency, displacement robustness, and scalability.

## 2. Structure and Methods

In recent years, significant progress has been made in broadband achromatic design, structural dispersion engineering, and meta-surface lens design methods based on optimization algorithms. For instance, Wang et al. established the phase compensation theory in their research on broadband achromatic meta-surface lenses, providing a theoretical foundation for subsequent optimization designs [33]; Chen et al. employed the finite-difference time-domain method and systematic parameter scanning to create a comprehensive “structure-phase” database, addressing the issue of insufficient structural units in the 0–π phase range [34]; Shrestha et al. innovatively applied the particle swarm optimization algorithm to the phase matching problem of meta-lens, establishing a six-dimensional optimization model to achieve collaborative optimization for multiple characteristic wavelengths [35]; Fan et al. combined the FDTD method with angular spectrum analysis to evaluate and validate the near-field characteristics and far-field performance of meta-surface lenses [34]. Approaches such as multi-structural unit phase libraries, deep learning-assisted structural inversion, and multi-scale simulation frameworks integrating FDTD and angular spectrum methods have gradually matured, providing a robust theoretical foundation for meta-surface lenses in broadband focusing and functional multiplexing [36,37,38]. In light of this, this study has developed a comprehensive design framework consisting of “phase library expansion—multi-wavelength collaborative optimization—structural interleaving fusion—multi-scale simulation verification.” Our team first designed hybrid parameter sweeps for square pillars, cylinders, and nanoring structures to expand the 0–π phase library, enhancing phase coverage capability for large-aperture lenses. Based on the wavelength compensation model and PSO, broadband achromatic design within the 1260–1360 nm range was achieved. Furthermore, an innovative structural interleaving method was proposed to spatially integrate phases for multi-scenarios (0, ±1 μm), significantly improving the lens displacement tolerance. Finally, a multi-scale simulation system combining FDTD and angular spectrum methods was constructed to efficiently verify both structural-level and lens-level performance. This approach not only unifies broadband, anti-lens-displacement perturbations and macro-scale design but also provides a systematic technical pathway for engineering high-performance meta-surface lenses.

### 2.1. Structural Interleaving and Angular Spectrum Method

**Figure 1:**
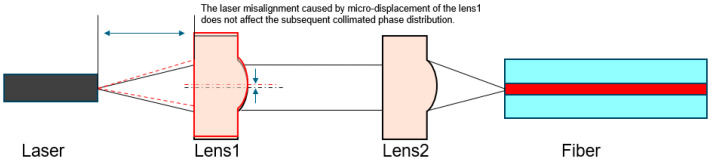
Schematic of Optical Path System and Actual Issues Faced. (The black solid line and dashed line represent the marginal ray and the principal optical axis when the lens optical path is well aligned; The red dashed line represent the marginal ray and the principal optical axis when the lens undergoes micro-displacement).

To achieve stable performance of the meta-lens under a “±1 μm” micro-displacement, this study innovatively proposes a “structural interweaving” design method and employs angular spectrum theory as a rapid evaluation tool. The core idea of the “structural interweaving” method is to independently optimized phase distributions $\phi_{A}(r)$, $\phi_{B}(r)$, and $\phi_{C}(r)$ for three operational conditions: the central position (0, 0), positive offset (0, +1 μm), and negative offset (0, −1 μm), as illustrated in Figure 1. An alternating interleaved arrangement strategy is utilized to combine microstructures of different functionalities, enabling the fusion of their respective phase distributions.

This method enables a single device to handle multiple operating states. To verify its performance, angular spectrum theory is introduced for a more faster and accurate far-field analysis. First, the output field is decomposed into an angular spectrum via two-dimensional Fourier transform.

(1) $$A(k_{x},k_{y};0)=\iint \;U_{\mathrm{out}\;}(x,y,0)exp[-i2\pi (k_{x}x+k_{y}y)]dxdy$$

where $k_{x}$ and $k_{y}$ are the spatial frequencies. The angular spectrum after propagation distance *z* is:

(2) $$A(k_{x},k_{y};z)=A(k_{x},k_{y};0)\times exp[ik_{z}z]$$

where $k_{z}=\frac{2\pi }{\lambda }\sqrt{1-{\left(\lambda k_{x}\right)}^{2}-{\left(\lambda k_{y}\right)}^{2}}$. Finally, the far-field distribution is obtained through inverse Fourier transform:

(3) $$U(x,y,z)=\iint \;A(k_{x},k_{y};z)exp[i2\pi (k_{x}x+k_{y}y)]dk_{x}dk_{y}$$

By analyzing the stability of ${\left|U(x,y,z)\right|}^{2}$ under various displacement conditions, the tolerance performance of a meta-lens can be quantitatively evaluated. This method significantly improves efficiency compared to full-wave simulations, providing an effective tool for large-scale optimization design.

### 2.2. PSO-Based Achromatic Metalens

Lenses exhibit frequency-dependent focusing effects on light of different frequencies, causing light of various wavelengths to fail to focus on the same plane. This often leads to the degradation of imaging performance in broadband imaging systems, resulting in chromatic aberration.

The chromatic aberration of traditional refractive lenses stems from the material’s refractive index dispersion, which can only be compensated by combining multiple lenses of different materials. This results in systems that are bulky, structurally complex, and costly. In contrast, as diffractive optical devices based on subwavelength units, the chromatic aberration of meta-lenses primarily arises from structural resonance dispersion and free-space propagation phase. However, their phase modulation mechanism is highly programmable, allowing for customized dispersion engineering on a single-layer planar device, inherently offering broadband achromatic potential [39,40]. Additionally, meta-lenses possess advantages such as ultrathin and lightweight designs, ease of integration, and low-cost manufacturing via mature nanofabrication processes, without the issue of multi-order energy loss found in traditional diffractive lenses. Therefore, the development of broadband achromatic meta-lenses not only significantly reduces system volume and complexity but also provides an important technological pathway for efficient and compact next-generation optical systems.

The phase distribution of a focusing meta-lens is as follows:

(4) $$\varphi (R,r)\;=\;-\frac{1}{\lambda }[2\pi \cdot (\sqrt{R^{2}\;+\;f^{2}}\;-\;f)]$$

where $R\;=\;\sqrt{x^{2\;}+\;y^{2}}$ is the distance from any point on the meta-lens to its center, and “f” is the focal length. To achieve an achromatic meta-lens, it is necessary to provide a phase delay that maintains a constant focal length across a broad wavelength range. Let $\lambda_{\max}$ and $\lambda_{\min}$ be the upper and lower bounds of the wavelength, respectively. Generally, for operation within a specific wavelength range, the formula can be expressed as:

(5) $$\varphi_{\mathrm{lens}}(R,\lambda )=\varphi_{\mathrm{lens}}(R,\lambda_{\max})\;+\;\Delta \varphi (R,r)$$

where

(6) $$\Delta \varphi (R,r)=\;-(\frac{1}{\lambda }\;-\;\frac{1}{\lambda_{\max}})[2\pi (\sqrt{R^{2}+f^{2}\;}-\;f)]$$

The broadband achromatic function can be achieved only when both the phase characteristics of the reference phase $\varphi_{\mathrm{lens}}(R,\;\lambda_{\max})$ and the phase compensation Δφ(R, r) are simultaneously satisfied [41]. The former manifests as a wavelength-independent phase modulation, which is solely related to $\lambda_{\max}$ and independent of the operating wavelength λ. The corresponding phase distribution can be obtained using the geometric phase principle, and this phase modulation mechanism depends only on the handedness of the unit structure. Conversely, the latter Δφ(R, r) is a function related to the operating wavelength and exhibits a linear relationship with 1/λ, representing the phase difference between different incident wavelengths. This phase difference can be compensated by designing the transmission phase response of each unit structure, and the phase response of the unit structure should also maintain a linear relationship with 1/λ. Due to the different mechanisms of transmission phase response and geometric phase, they can be designed to not interfere with each other and can be simply combined. However, not every unit structure’s transmission phase has a linear relationship with 1/λ, so an additional phase factor C(λ) can be introduced to achieve phase compensation at specific wavelengths through optimized design. It is noteworthy that such phase shifts do not affect the focusing characteristics of the meta-lens. Based on this, the phase profile of the achromatic meta-lens can be rewritten as:

(7) $$\varphi (R,r)=\;-\frac{1}{\lambda }[2\pi \cdot (\sqrt{R^{2}\;+\;f^{2}}\;-\;f)]\;+\;C(\lambda )$$

where C(λ) is a phase factor dependent solely on wavelength, obtained through an optimization algorithm.

### 2.3. Numerical Simulation and Computational Methods

This study employs a multi-scale, multi-physics numerical simulation approach, systematically characterizing the optical performance of meta-lenses through a strategy combining the Finite-Difference Time-Domain (FDTD) method with angular spectrum theory. At the nanoscale, based on Maxwell’s equations, a three-dimensional FDTD method is used for rigorous electromagnetic simulations of meta-atom units. Through parameter scanning, a comprehensive “structure-phase-dispersion” response database is constructed, with its governing equations represented as

(8) ∇×H=ε∂E/∂t

(9) ∇×E=−μ∂H/∂t

At the device-scale, the far-field distribution of the metalens is efficiently calculated using angular spectrum theory:

(10) $$U(x,y,z)=\iint A(f_{x},f_{y};0)\cdot \exp[-i2\pi (f_{x}x+f_{y}y)]df_{x}df_{y}$$

This multi-scale hybrid computational method ensures both an accurate description of electromagnetic interactions at the nanoscale and rapid evaluation of far-field performance for large-scale meta-lenses. The FDTD simulations adopt a 5 nm mesh precision to guarantee computational accuracy, while angular spectrum theory reduces the far-field analysis time for large-scale lenses from several days to a few minutes, providing a reliable numerical experimental platform for the optimized design of meta-lenses.

## 3. Simulation and Discussion

This study addresses the sensitivity of the lens-fiber coupling system to chromatic aberration and micro-displacement, proposing an achromatic meta-lens design based on particle swarm optimization algorithm and structural interleaving method. The effectiveness of the structural interleaving and particle swarm optimization algorithm was preliminarily verified by constructing micro-scale small lens structures, solving the problem of excessive computational resources required for FDTD simulation of macroscopic lenses. Subsequently, based on the small lens design, the micro-scale small lens structure was extended to a macroscopic large lens, and the off-axis collimation characteristics and achromatic performance in the 1260~1360 nm optical band were evaluated using angular spectrum theory. This approach not only effectively enhances the lens’s chromatic aberration suppression capability and alignment tolerance over a broad bandwidth but also employs a collaborative design framework of structural interleaving and optimization algorithms with strong universality. It provides a reliable design method for further developing high-performance, low-sensitivity, and easily integrable on-chip optical coupling systems, and is applicable to other micro-optical system designs requiring wideband achromatic performance and high alignment stability.

### 3.1. Simulation Design Verification of Off-Axis Achromatic Micro-Scale Lenses

The micro-lens simulation requires low computational resources and offers fast iteration speeds, making it an ideal platform for verifying method effectiveness and optimizing processes. By designing a small-sized (D = 31 μm) meta-lens, the feasibility of achieving achromatic characteristics based on the particle swarm optimization (PSO) algorithm and the collimation design with structural interleaving for resistance to micro-displacement is verified.

#### 3.1.1. Achromatic Function Validation of Small Lens

**Figure 2:**
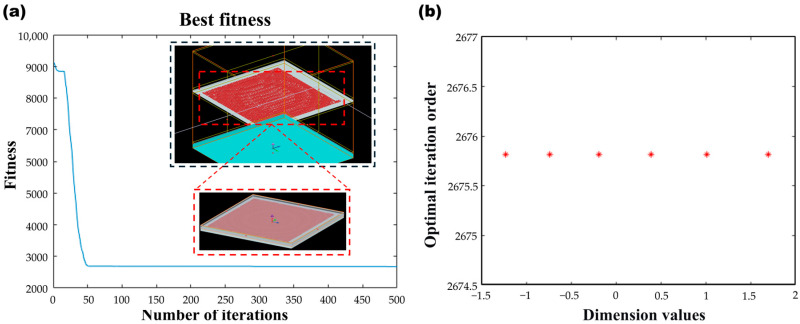
Convergence curve of fitness values during the PSO optimization process (**a**) convergence curve of fitness values and the FDTD model of the composite collimating lens; (**b**) the iteration order of different dimensions in the particle swarm optimization algorithm. (Within the dashed line are the lens structure diagram from the FDTD model and its local enlarged schematic).

To address the issue of achromatism in the O-band, this study employs the Finite-Difference Time-Domain (FDTD) method for rigorous electromagnetic simulations. The initial microstructure nano-pillar is designed as a rectangular shape to provide degrees of freedom for two polarization directions. Its length and width are parameter-swept within a range of 50 nm to 500 nm, while the height is fixed at 800 nm to provide sufficient phase modulation depth, as shown in Figure 2a. The simulated wavelength range covers 1260 nm to 1360 nm. The parameter sweeps results indicate a complex nonlinear relationship between the transmission phase of the nano-pillar and its geometric dimensions. Although this structure can achieve a phase coverage close to 2π at specific wavelengths, its phase distribution is inconsistent across different wavelengths. Moreover, within the phase range of 0 to π, the number of available structural units is relatively limited. This insufficiency in phase coverage, coupled with mismatched dispersion characteristics, is the fundamental reason why a single structure struggles to achieve broadband achromatism. To resolve the phase-matching challenges described above, the design of the achromatic meta lens is transformed into a multi-objective optimization problem. The target lens diameter is set to D = 31 μm, the focal length to F = 15.5 μm, and the light source is located at point (0, 0). To achieve broadband achromatism, the problem is modeled as a six-dimensional optimization problem, and the Particle Swarm Optimization (PSO) algorithm is applied to optimize the phase compensation factor C(λ). Through multiple iterations, as shown in Figure 2b, the convergence of its fitness value is investigated.

**Figure 3:**
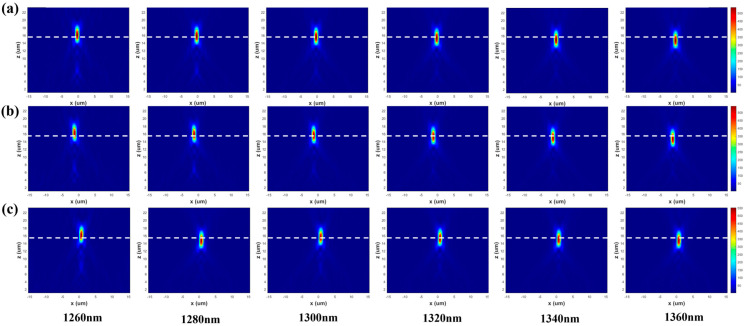
6 wavelength focusing effects ((**a**). when the light source is at (0, 0); (**b**). when the light source is at (−1, 0); (**c**). when the light source is at (+1, 0), the dashed line represents a focal length of 15.5 μm).

By randomly selecting 6 wavelengths within the design band for focusing simulation, the performance of the optimized lens was objectively evaluated. As shown in Figure 3, the simulation results clearly indicate that the light fields of all 6 wavelengths successfully converged, with the focal positions densely distributed around a focal length of 15.5 μm. The simulation data show that the standard deviation of the deviation between the focal points of each wavelength and the design focal length is less than 0.5 μm, which is significantly smaller than that of traditional diffractive lenses. This quantitative result strongly demonstrates the effectiveness of the PSO algorithm design and the excellent achromatic performance of the meta-lens within the 100 nm bandwidth.

#### 3.1.2. Off-Axis Performance Verification of Small Lenses

**Figure 4:**
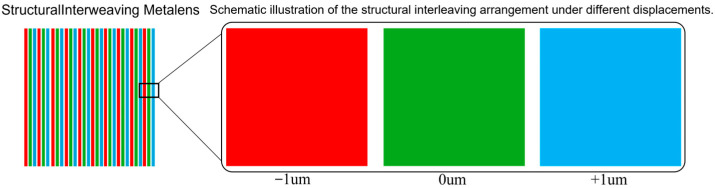
The strategy of interleaved fusion of different structures (Different colors represent the microstructures corresponding to different offsets).

In practical optoelectronic packaging, lenses may experience micro-displacements of approximately ±0.5 μm due to adhesive curing shrinkage or environmental temperature variations. Therefore, this study validates the collimation performance when the point source is positioned at the optical axis focus (0, 0), the positive off-axis position 1 μm above the vertical optical axis focus (0, 1), and the negative off-axis position 1 μm below the vertical optical axis focus (0, −1). Additionally, the design must achieve good achromatic performance within the 1260–1360 nm range. For off-axis collimation, the key is to ensure that the angle and displacement of the collimated beam remain unchanged, thereby maintaining the optical path after the collimating lens and ensuring consistent system insertion loss. Here, we innovatively propose a “structural interweaving” method, which spatially multiplexes and integrates the phase distributions of the central lens (0, 0), the off-axis lens (0, −1), and the off-axis lens (0, 1). By customizing an FDTD script, the three phase profiles are fused to generate a composite meta-surface structure. The strategy of interleaved fusion of different structures is illustrated in Figure 4.

**Figure 5:**
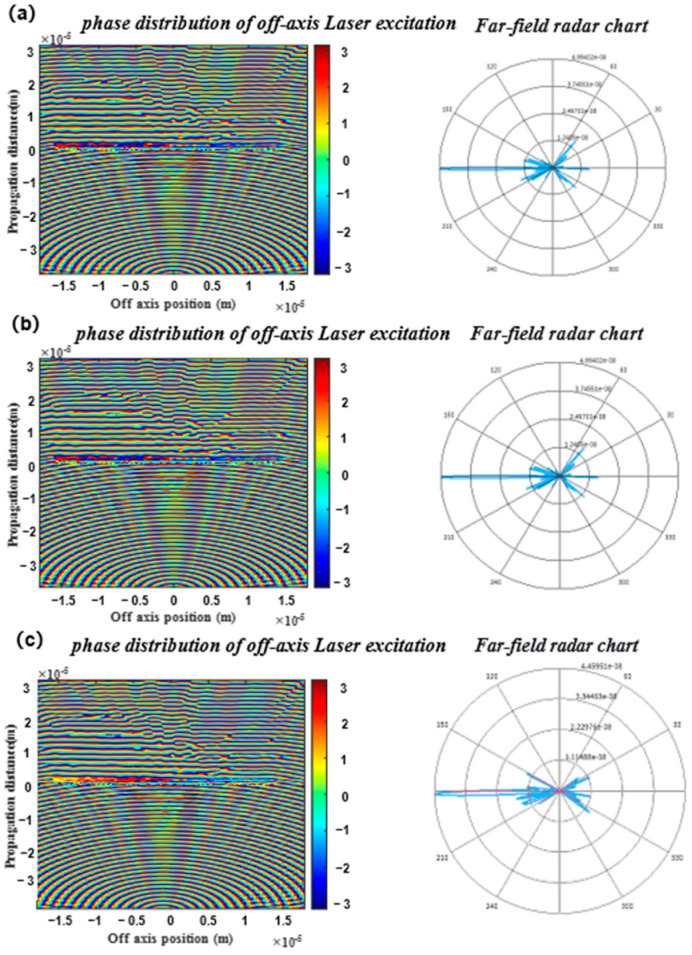
Collimating lens verification ((**a**), cross-sectional phase and far-field distribution of the origin collimating lens; (**b**), cross-sectional phase and far-field distribution of the +1 μm collimating lens; (**c**), cross-sectional phase and far-field distribution of the −1 μm collimating lens).

Simulation results show that when the point source is at (0, 0), as illustrated in Figure 5a, the spherical wave phase distribution of the point source is transformed into a phase distribution parallel to the lens plane after passing through the lens, indicating that the light field changes from divergent to uniform collimated light. The far-field pattern exhibits a regular circular spot with a concentrated main lobe, meeting the expected collimation performance. When the off-axis displacement is +1 μm, as shown in Figure 5b, the corresponding sub-region dominates the modulation. The phase distribution before and after the meta-lens indicates that the light field remains collimated, and the far-field pattern displays a regular spot with slight offset and deformation but a sharp main lobe. When the off-axis displacement is −1 μm, as depicted in Figure 5c, the mirror-symmetric sub-region is activated. The phase distribution before and after the meta-lens confirms that the light field remains collimated, and the far-field pattern is symmetric to that of the +1 μm case. Finally, the three structures are combined into a composite system, producing uniform and seamless collimated spots. The far-field performance is nearly ideal with concentrated energy, demonstrating that this design achieves high-quality and stable collimation within a ±1 μm displacement range, highlighting its strong potential for engineering applications.

### 3.2. Simulation of Achromatic Macro Lense

This study begins with unit cell structure simulation, constructing a high-density nanostructure phase library through joint scanning of multiple structural and multidimensional parameters to meet the wide phase coverage and achromatic requirements of large-aperture lenses. Based on this, combined with theoretical target phase distribution, the particle swarm optimization algorithm is employed to match the optimal structure for each pixel from the phase library, achieving high-precision approximation of the actual phase to the ideal phase. Finally, the collimation and focusing performance of the designed lens is verified through angular spectrum optical simulation. The results demonstrate that this method can effectively support the design of large-radius achromatic meta-lens and achieve excellent focusing outcomes.

#### 3.2.1. Macro-Scale Lens Simulation Verification Results

**Figure 6:**
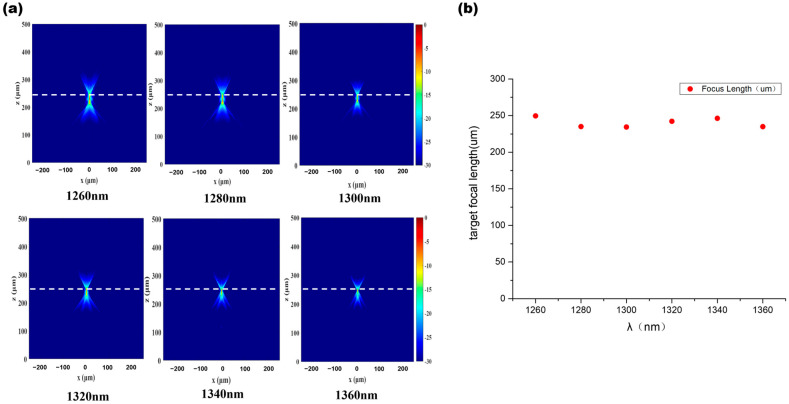
Large Lens Achromatic Effect ((**a**). Focusing effect diagrams of different wavelengths, the dashed line represents a focal length of 250 μm; (**b**). Focal length data distribution).

After validating the applicability of the research methodology using a small-scale lens, the approach was extended to the design of a macro lens suitable for the product optical path illustrated in Figure 1. Specifically, a metalens structure with a radius of 250 μm and a focal length of 250 μm was designed for operation in the 1260–1360 nm wavelength band. The key results are presented and discussed in detail. Due to the excessively large radius of the primary lens, it is impossible to simulate its results in FDTD to achieve propagation and expansion from the micro-lens to the primary lens. Therefore, this study employs the angular spectrum method for far-field prediction. The angular spectrum method decomposes the exit field through Fourier analysis, representing it as an angular spectrum of plane waves, and uses the propagation transfer function to calculate the field distribution at any distance. As shown in Figure 6, the focal light field distribution results calculated using the angular spectrum method clearly indicate that the focal positions for different wavelengths fluctuate around approximately 250 μm, achieving a relatively good achromatic effect.

**Figure 7:**
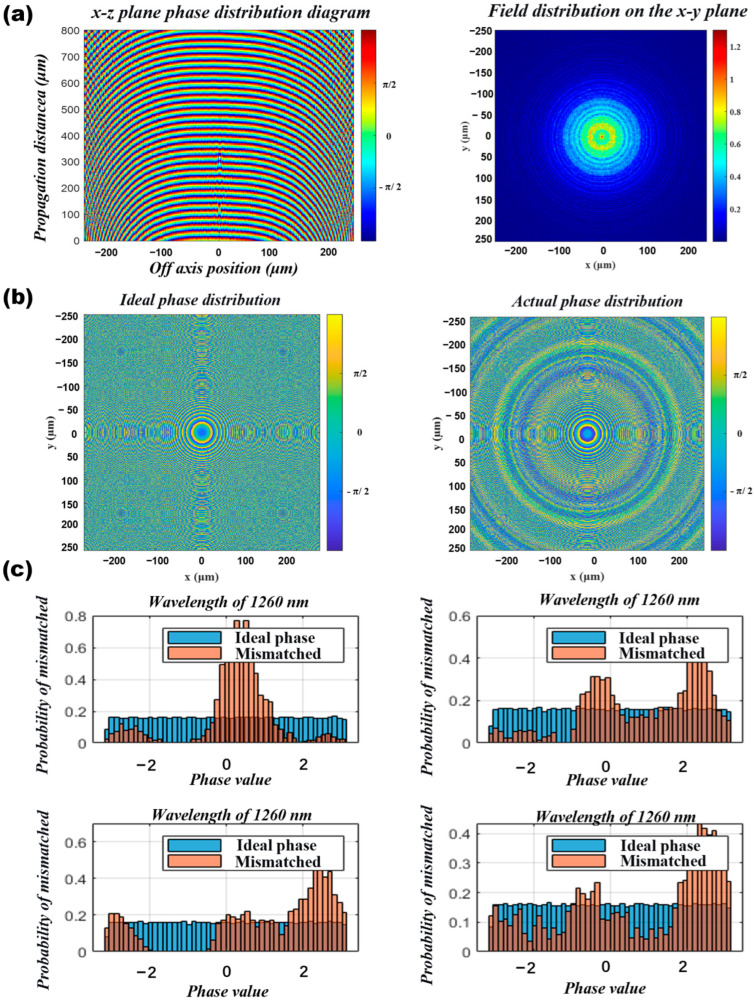
Macro Lens Collimation Test and Phase Contrast ((**a**). Point Source Collimation Verification—Phase Distribution and Light Field Distribution; (**b**). Ideal Phase vs. Actual Phase Results Comparison; (**c**). Phase Database vs. Ideal Phase Comparison).

Similarly, the collimation characteristics of a point source using the large lens were evaluated using the angular spectrum method. The results, as shown in Figure 7a, indicate that although the overall collimation effect of the spot is acceptable, the central morphology and field distribution are poor, presenting higher-order mode distributions that fail to meet the requirements for spot quality in practical engineering applications. To investigate the cause of the unsatisfactory collimation effect, we compared the differences between the ideal and actual phase distributions, revealing a significant deviation between the actual and ideal phases, as illustrated in Figure 7b. To further examine the issue of phase deficiency, we retrieved the structural and phase information from the parameter scanning sample library and compared it with the ideal phase requirements. The analysis showed that the database contains very few structural units corresponding to the 0–π phase range, as seen in Figure 7c. In summary, during the empirical design of large lenses based on small lens experience, insufficient structural phase data and suboptimal phase compensation have emerged as significant problems.

#### 3.2.2. Segmental Scanning for Database Extension

To enhance the diversity of the database, this study employs a segmented parameter sweep on the structures. The segmented sweep process focuses on square and cylindrical structures, beginning with a segmented sweep of the square structure: In the first step, square pillar dimensions of 50–100 nm were tested, revealing that the color bar values (−2.25 to −2.16) were all negative, unable to provide a phase greater than 0. In the second step, the square pillar dimensions were expanded to 100–200 nm, yet the sweep results still yielded entirely negative phases (−2.1 to −1.4). In the third step, the square pillar dimensions were further increased to 200–300 nm, at which point the color bar displayed most regions in green to red (corresponding to positive phases of 0–π), meeting the requirement for positive phases for the first time. Consequently, the number of phase units within this effective range (200–300 nm) was expanded. Building on this, cylindrical structures were introduced, with their radius range set to 25–300 nm for a new round of parameter sweeps. It can be observed that this novel structure provides unique phase response characteristics within specific dimensional ranges.

**Figure 8:**
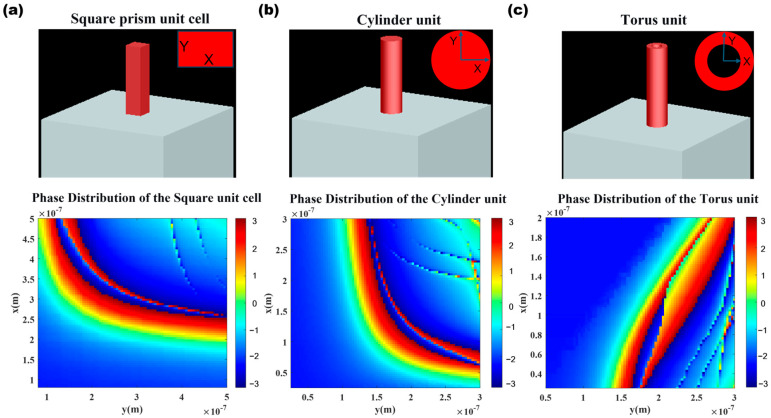
Segmented Sweep Parameter Data Verification ((**a**), sweep results for square pillars 50 nm~100 nm, the X and Y axes correspond to the long and short sides respectively; (**b**), sweep results for cylinders 200 nm~300 nm, the X and Y axes correspond to the radii in the two directions respectively; (**c**), Sweep results for rings with inner diameter 25 nm~200 nm, outer diameter 50 nm~300 nm, the X and Y axes correspond to the inner and outer diameters of the ring).

To further enhance performance, we have expanded the scale of the database on the existing foundation by introducing toroidal nanostructures. The inner diameter of the toroidal structure varies from 25 nm to 200 nm, while the outer diameter ranges from 50 nm to 300 nm. The rich geometric parameters provide new degrees of freedom for achieving more precise phase control. Figure 8c illustrates the geometric morphology of the toroidal structure and the results of its parameter scanning, revealing that this new structure offers unique phase response characteristics within specific size ranges and enables more accurate phase matching outcomes.

#### 3.2.3. Performance Verification of the Optimized Meta-Lens Structure

**Figure 9:**
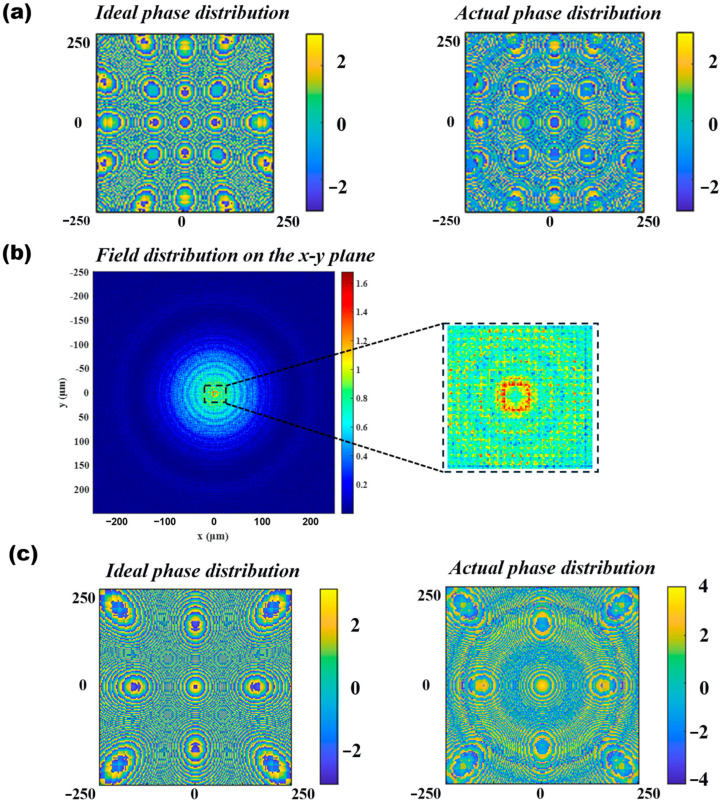
Verification comparison across different sample sizes ((**a**), comparison between ideal phase and actual phase; (**b**), first optimization of collimating lens effect test; (**c**), comparison of optimized ideal phase results with actual phase).

Through the systematic expansion of the unit phase database, we reapplied the Particle Swarm Optimization (PSO) algorithm to optimize the phase distribution of the lens for better matching. As shown in Figure 9a, it can be observed that the error between the actual phase and the ideal distribution is significantly reduced, the phase deviation in the edge regions is effectively corrected, and the continuity is markedly improved. Moreover, based on the optimization results, the algorithm reasonably selected the most suitable nanostructure types in different areas, compensating for the insufficient phase modulation capability of a single structure type. Further analysis of the lens’s phase profile in the x/y-plane reveals that the database expansion improved the agreement between the actual phase and the ideal contour, resulting in smoother curves, thereby laying the foundation for high-quality wavefront control. We further conducted preliminary tests on the collimation performance of the lens. As shown in Figure 9b, although the focal spot shape of the beam still exhibits some distortion, it has been significantly improved compared to the pre-optimization state, though further refinement is still required. Therefore, we continued to expand the database and introduced ring-shaped structures (inner diameter 25–200 nm, outer diameter 50–300 nm). Leveraging their unique phase response characteristics and after a second round of PSO, the phase error was further reduced. As shown in Figure 9c, particularly in regions of rapid variation, the matching accuracy was significantly enhanced, demonstrating the advantage of using mixed structure types—different geometric structural units played complementary roles in phase modulation [42]. Subsequently, the updated structural blueprint shows that the rings and the original structures exhibit excellent functional complementarity, with the phase profile displaying smooth and continuous characteristics.

**Figure 10:**
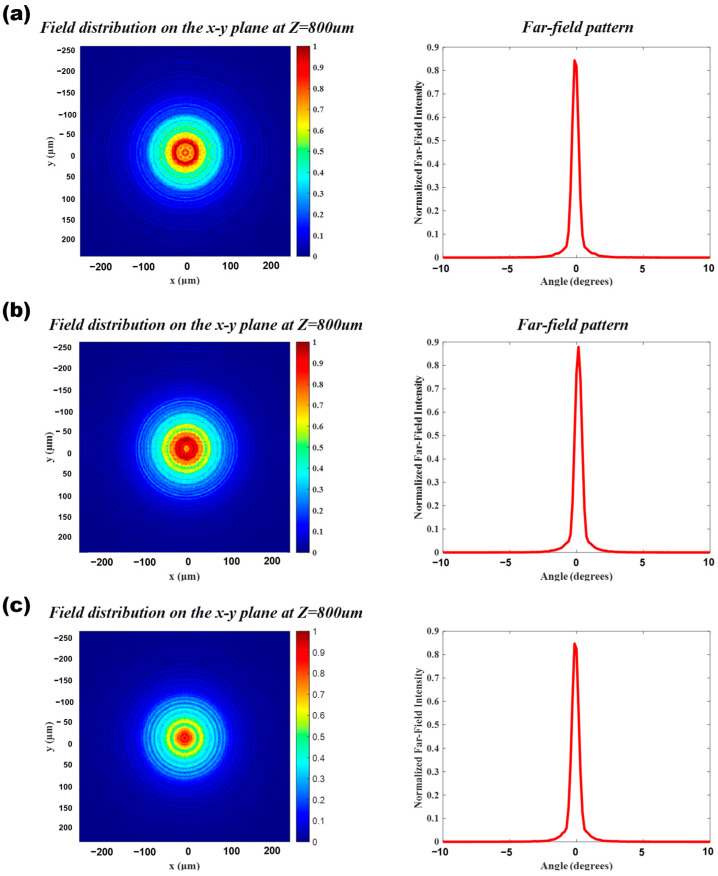
Collimated spot and far-field patterns of the optimized meta-lens at different positions: (**a**) on-axis, (**b**) +1 μm off-axis, (**c**) −1 μm off-axis.

The final collimation performance of the twice-optimized lens was outstanding. As seen in the far-field spot and beam pattern diagrams in Figure 10a, the spot exhibits a Gaussian distribution with a sharp main lobe and a small divergence angle, validating the effectiveness of the multi-structure hybrid strategy. Combining systematic database expansion with optimization algorithms can significantly enhance the phase control accuracy and beam quality of large-radius meta-lenses.

**Figure 11:**
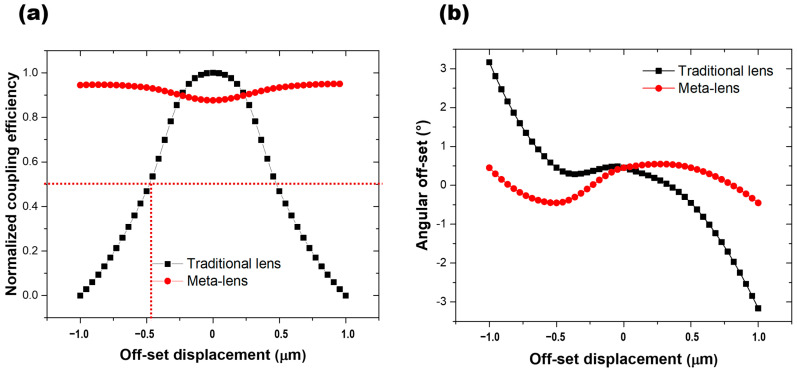
Comparison of tolerance between the metalens and the conventional lens. (**a**) Tolerance in the direction perpendicular to the optical axis. (**b**) Collimation angular deviation under different lateral displacements perpendicular to the optical axis.

Furthermore, we have calculated the focusing efficiency as a function of the lateral displacement of the metalens central axis. The results indicate that the focusing efficiency exhibits minimal variation with increasing displacement, whereas for a conventional lens structure, the focusing efficiency decreases sharply as the displacement increases. Taking the displacement at which the efficiency drops to 50% of its maximum value as the tolerance limit, the conventional lens exhibits a tolerance range of only 0.5 μm, while the metalens maintains a high coupling efficiency over the entire range from −1 μm to +1 μm, as shown in the Figure 11a.

The metalens is designed as a collimating lens, and its collimation performance can be characterized by the angular deviation of the outgoing beam from the optical axis, as shown in the Figure 11b. For the metalens, the angular deviation remains within 0.5° across the displacement range, whereas the conventional lens exhibits significantly larger deviations, exceeding 3° at large displacements.

Comparing the absolute performance of the two structures, the peak intensity of the metalens at zero displacement is indeed slightly lower. However, the overall uniformity and the tolerance range are substantially improved.

Although the metalens is composed of three interleaved regions, the modulation effect remains continuous for positions between these interleaved segments without exhibiting any pronounced discontinuities. This is because the structural unit size of the metalens is significantly smaller than the operating wavelength, and its effective refractive index or phase response varies smoothly and continuously in space. Even with the spatial interleaving of different regions, the overall phase modulation function remains smooth, thereby preventing the emergence of obvious discontinuous features. Furthermore, as is typical for diffractive optical elements such as metalenses, constraints are imposed on the relationship between the unit cell spacing and the wavelength to ensure that higher-order diffraction orders remain effectively suppressed.

Table 1 provides a detailed comparison of key performance parameters of the meta-lens before and after optimization across different wavelengths. As clearly shown in the table, after optimization with the PSO algorithm, the standard deviation of the focal lengths corresponding to each wavelength significantly decreased from an initial 3.2 μm to 0.4 μm, and the focal length fluctuation rate improved from ±10.3% to ±1.3%, fully validating the effectiveness of the proposed phase compensation method. In terms of phase matching accuracy, the root means square error (RMSE) after optimization decreased from 0.82 rad to 0.15 rad, and the phase matching efficiency increased from 78.5% to 95.2%. This indicates that by introducing the wavelength-dependent phase factor C(λ), the limitations of traditional linear phase compensation methods in handling nonlinear dispersion have been successfully addressed. Notably, with the introduction of the “structural interleaving” method, the performance stability of the system within a ±1 μm displacement range has been significantly improved. The coupling efficiency fluctuation decreased from >30% before optimization to <8%, and the beam quality factor M^2^ for all wavelengths remained below 1.2, demonstrating the advantages of this method in maintaining beam quality.

### 3.3. Off-Axis Meta-Lens Point Source Displacement Robustness Test

The previous text established a microstructure of micro-lenses, preliminarily verifying the effectiveness of structural interpenetration and the particle swarm optimization algorithm. This section focuses on scaling up the microstructure of micro-lenses to a macroscale large lens, repeating the research approach used for the micro-lenses. By employing angular spectrum theory and blending three types of phases at a specific ratio, a composite meta-surface structure is generated to achieve the design of a displacement-resistant large lens.

To verify the robustness and adaptability of the designed lens to changes in the light source position, the study comprehensively investigated its phase-matching accuracy and optical performance by applying ±1 μm displacement excitation to the point source, as shown in Figure 10. First, under the +1 μm displacement, the actual and ideal phase distributions were in overall high agreement, with only minor local deviations, indicating that the phase database possesses strong generalization capability. The structural blueprint revealed that units such as square columns, cylinders, and rings were arranged with radial symmetry, with a continuous and smooth phase profile and stable wavefront modulation capability. Furthermore, in the collimation test, the far-field spot morphology was regular and energy was concentrated (with only slight asymmetric distortion). Simulations using the angular spectrum method confirmed that the far-field expansion was small, the main lobe was strong, and the side lobes were well suppressed, meeting the requirements for parallelism and energy concentration. Next, the light source was set to a −1 μm reverse displacement to examine the lens’s response characteristics under reverse offset. Comparative analysis revealed that the phase error was comparable to that under +1 μm displacement, and the structural blueprint and phase profile remained reasonably smooth. The collimated spot quality was similar, free of aberrations, and far-field simulations showed a compact spot with a sharp main lobe, confirming the lens’s symmetrical performance and structural robustness. Finally, the baseline and ±1 μm offset-optimized structures were combined into a composite lens system. Collimation tests revealed a uniform spot with no visible splicing traces, while far-field inspection showed a nearly ideal spot with concentrated energy and a small divergence angle. This demonstrates that the system can still output high-quality parallel light under complex integration, meeting the stringent engineering requirements for collimation performance.

This study establishes a systematic design workflow for large-scale meta-lenses. Starting from validation on a small-scale lens, the design proceeds to aperture scaling using the angular spectrum method, followed by phase library expansion and sample size optimization. The resulting large-scale meta-lens maintains excellent collimation performance and far-field quality even under a ±1 μm light source offset, demonstrating superior robustness and strong potential for engineering applications.

## 4. Conclusions

In summary, this paper presents and systematically validates a meta-lens design that simultaneously achieves broadband achromatic performance and high displacement tolerance for laser–lens–fiber coupling systems. Two key innovations address the core challenges. First, a structural interleaving strategy integrates phase profiles optimized for 0 and ±1 μm incident offsets into a single meta surface. This enables adaptive wavefront compensation upon source displacement, significantly enhancing system robustness. Second, a hybrid phase database constructed from multiple nanostructure types (square pillars, cylinders, and rings) expands phase coverage density within the 0–2π range, resolving the phase discretization issue inherent in large-aperture meta-lenses. Combined with particle swarm optimization and multi-scale simulation, the design achieves achromatic performance across the 1260–1360 nm band, with phase matching efficiency exceeding 95% and stable collimation maintained within ±1 μm displacement. The results validate the effectiveness and engineering feasibility of the proposed approach for large-scale, high-robustness meta-lens fabrication.

Future work will advance the meta-lens toward engineering and intelligentization. First, constructing a larger-scale, CMOS-compatible phase library will enhance large-area fabrication consistency and mass production capability. Second, data-driven methods such as deep learning will accelerate structural inversion and multi-objective optimization for more efficient broadband and anti-displacement designs. Furthermore, integrating phase-change materials or tunable structures may enable adaptive meta-lenses with dynamic focal adjustment and real-time displacement compensation, providing stable and intelligent optical solutions for high-speed communication, optoelectronic packaging, and integrated photonic systems.

## Figures and Tables

**Table 1:** Comparative performance parameters of achromatic meta lenses.

Performance Metrics	Pre-Optimization	Post-Optimization	Improvements
Focal length standard deviation (μm)	3.2	0.4	87.5%
Focal length volatility	±10.3%	±1.3%	87.4%
Phase Matching RMSE (rad)	0.82	0.15	81.7%
Phase matching efficiency	78.5%	95.2%	21.3%
±1 μm displacement coupling efficiency fluctuation	>30%	<8%	>73.3%

## Data Availability

The data presented in this study are available upon request from the corresponding authors.

## References

[1] Ou K., Wan H., Wang G., Zhu J., Dong S., He T., Yang H., Wei Z., Wang Z., Cheng X. (2023). Advances in meta-optics and metasurfaces: Fundamentals and applications. Nanomaterials.

[2] Hsu W.L., Chen Y.C., Yeh S.P., Zeng Q.C., Huang Y.W., Wang C.M. (2022). Review of metasurfaces and metadevices: Advantages of different materials and fabrications. Nanomaterials.

[3] Hu Z., Gu M., Tian Y., Li C., Zhu M., Zhou H., Fang B., Hong Z. (2025). Review for optical metalens based on metasurfaces: Fabrication and applications. Microsyst. Nanoeng..

[4] Colburn S., Zhan A., Majumdar A., Majumdar A. (2018). Metasurface optics for full-color computational imaging. Sci. Adv..

[5] Ren H., Jang J., Li C., Aigner A., Plidschun M., Kim J., Rho J., Schmidt M.A., Maier S.A. (2022). An achromatic metafiber for focusing and imaging across the entire telecommunication range. Nat. Commun..

[6] Yuan G., Rogers E., Zheludev N. (2017). Achromatic super-oscillatory lenses with sub-wavelength focusing. Light Sci. Appl..

[7] Khorasaninejad M., Chen W.T., Devlin R.C., Oh J., Zhu A.Y., Capasso F. (2016). Metalenses at visible wavelengths: Diffraction-limited focusing and subwavelength resolution imaging. Science.

[8] Bowen P.T., Baron A., Smith D.R. (2017). Effective-medium description of a metasurface composed of a periodic array of nanoantennas coupled to a metallic film. Phys. Rev. A.

[9] Fan Q., Huo P., Wang D., Liang Y., Yan F., Xu T. (2017). Visible light focusing flat lenses based on hybrid dielectric-metal metasurface reflector-arrays. Sci. Rep..

[10] Wesemann L., Rickett J., Davis T.J., Roberts A. (2022). Real-time phase imaging with an asymmetric transfer function metasurface. ACS Photonics.

[11] Chen W.T., Zhu A.Y., Sanjeev V., Khorasaninejad M., Shi Z., Lee E., Capasso F. (2018). A broadband achromatic metalens for focusing and imaging in the visible. Nat. Nanotechnol..

[12] Arbabi A., Horie Y., Bagheri M., Faraon A. (2015). Dielectric metasurfaces for complete control of phase and polarization with subwavelength spatial resolution and high transmission. Nat. Nanotechnol..

[13] Richardson D.J., Fini J.M., Nelson L.E. (2013). Space-division multiplexing in optical fibres. Nat. Photonics.

[14] Sillard P., Bigot-Astruc M., Molin D. (2014). Few-mode fibers for mode-division-multiplexed systems. J. Light. Technol..

[15] Wallner O., Winzer P.J., Leeb W.R. (2002). Alignment tolerances for plane-wave to single-mode fiber coupling and their mitigation by use of pigtailed collimators. Appl. Opt..

[16] Nicia A. (1981). Lens coupling in fiber-optic devices: Efficiency limits. Appl. Opt..

[17] Yu N., Genevet P., Kats M.A., Aieta F., Tetienne J.P., Capasso F., Gaburro Z. (2011). Light propagation with phase discontinuities: Generalized laws of reflection and refraction. Science.

[18] Jiang J., Fan J.A. (2019). Global optimization of dielectric metasurfaces using a physics-driven neural network. Nano Lett..

[19] Wang Z., Li T., Soman A., Mao D., Kananen T., Gu T. (2019). On-chip wavefront shaping with dielectric metasurface. Nat. Commun..

[20] Zhang Y., Pu M., Jin J., Lu X., Guo Y., Cai J., Zhang F., Ha Y., He Q., Xu M. (2022). Crosstalk-free achromatic full Stokes imaging polarimetry metasurface enabled by polarization-dependent phase optimization. Opto-Electron. Adv..

[21] Chen W.T., Yang K.Y., Wang C.M., Huang Y.W., Sun G., Chiang I.D., Liao C.Y., Hsu W.L., Lin H.T., Sun S. (2014). High-efficiency broadband meta-hologram with polarization-controlled dual images. Nano Lett..

[22] Arbabi A., Horie Y., Ball A.J., Bagheri M., Faraon A. (2015). Subwavelength-thick lenses with high numerical apertures and large efficiency based on high-contrast transmitarrays. Nat. Commun..

[23] Goodman J.W. (2005). Introduction to Fourier Optics.

[24] Park J.S., Lim S.W.D., Amirzhan A., Kang H., Karrfalt K., Kim D., Leger J., Urbas A., Ossiander M., Li Z. (2024). All-Glass 100 mm Diameter Visible Metalens for Imaging the Cosmos. ACS Nano.

[25] Chang S., Zhang L., Duan Y., Rahman M.T., Islam A., Ni X. (2024). Achromatic metalenses for full visible spectrum with extended group delay control via dispersion-matched layers. Nat. Commun..

[26] Hao F., Zhao C., Zhang Y., Chen J., Li S., Zhou W., Ran C., Zeng Y., Chen H., He X. (2025). Centimeter-size achromatic metalens in long-wave infrared. Nanophotonics.

[27] Ou K., Yu F., Li G., Wang W., Chen J. (2021). Broadband achromatic metalens in mid-wavelength infrared. Laser Photonics Rev..

[28] Xie L., Wan H., Ou K., Long J., Wang Z., Wang Y., Yang H., Wei Z., Wang Z., Cheng X. (2024). High-Efficiency Broadband Achromatic Metadevice for Spin-to-Orbital Angular Momentum Conversion of Light in the Near-Infrared. Small Sci..

[29] Ma Z., Yan W., Qiu M. (2025). Dispersion Design Method of Metasurface Based on Semi-Inverse Phase Matching. Laser Photonics Rev..

[30] Chen Y.C., Hsu W.L., Zeng Q.C., Yu C.Y., Chen P.D., Chen C.C., Lin Y.H., Chen F.Z., Wang C.M. (2024). Broadband achromatic thermal metalens with a wide field of view based on wafer-level monolithic processes. Appl. Phys. Lett..

[31] Chen M., Cai J., Sun W., Chang L., Xiao X. (2018). High-Efficiency All-Dielectric Metasurfaces for Broadband Polarization Conversion. Plasmonics.

[32] Pan M., Fu Y., Zheng M., Chen H., Zang Y., Duan H., Li Q., Qiu M., Hu Y. (2022). Dielectric metalens for miniaturized imaging systems: Progress and challenges. Light Sci. Appl..

[33] Wang S., Wu P.C., Su V.C., Lai Y.C., Chen M.K., Kuo H.Y., Chen B.H., Chen Y.H., Huang T.T., Wang J.H. (2018). A broadband achromatic metalens in the visible. Nat. Nanotechnol..

[34] Fan Z.B., Qiu H.Y., Zhang H.L., Pang X.N., Zhou L.D., Liu L., Ren H., Wang Q.H., Dong J.W. (2019). A broadband achromatic metalens array for integral imaging in the visible. Light Sci. Appl..

[35] Shrestha S., Overvig A.C., Lu M., Stein A., Yu N. (2018). Broadband achromatic dielectric metalenses. Light Sci. Appl..

[36] Kwon H., Arbabi E., Kamali S.M., Faraji-Dana M.S., Faraon A. (2020). Single-shot quantitative phase gradient microscopy using a system of multifunctional metasurfaces. Nat. Photonics.

[37] He T., Liu T., Xiao S., Wei Z., Wang Z., Zhou L., Cheng X. (2022). Perfect anomalous reflectors at optical frequencies. Sci. Adv..

[38] Ming Y., Intaravanne Y., Ahmed H., Kenney M., Lu Y.Q., Chen X. (2022). Creating composite vortex beams with a single geometric metasurface. Adv. Mater..

[39] Hu Y., Jiang Y., Zhang Y., Yang X., Ou X., Li L., Duan H. (2023). Asymptotic dispersion engineering for ultra-broadband meta-optics. Nat. Commun..

[40] Wang X., Liu S., Xu L., Cao Y., Tao Y., Chen Y., Zhang Z., Chen C., Wang C., Ni J. (2024). A A holographic broadband achromatic metalens. Laser Photonics Rev..

[41] Yao J., Lin R., Che X., Fan Y., Liang Y., Zhang J., Chen M.K., Tsai D.P. (2023). Integrated-resonant units for phase compensation and efficiency enhancements in achromatic meta-lenses. ACS Photonics.

[42] Ou K., Yu F., Li G., Wang W., Miroshnichenko A.E., Huang L., Wang P., Li T., Li Z., Chen X. (2020). Mid-infrared polarization-controlled broadband achromatic metadevice. Sci. Adv..

